# Evidence of a pan-tissue decline in stemness during human aging

**DOI:** 10.18632/aging.205717

**Published:** 2024-04-04

**Authors:** Gabriel Arantes dos Santos, Gustavo Daniel Vega Magdaleno, João Pedro de Magalhães

**Affiliations:** 1 Laboratory of Medical Investigation (LIM55), Urology Department, Faculdade de Medicina FMUSP, Universidade de Sao Paulo, Sao Paulo 01246 903, Brazil; 2 Genomics of Ageing and Rejuvenation Lab, Institute of Inflammation and Ageing, University of Birmingham, Birmingham B15 2WB, United Kingdom; 3 Institute of Life Course and Medical Sciences, University of Liverpool, Liverpool L7 8TX, United Kingdom

**Keywords:** longevity, stem cells, transcriptomics, senescence

## Abstract

Despite their biological importance, the role of stem cells in human aging remains to be elucidated. In this work, we applied a machine learning methodology to GTEx transcriptome data and assigned stemness scores to 17,382 healthy samples from 30 human tissues aged between 20 and 79 years. We found that ~60% of the studied tissues exhibit a significant negative correlation between the subject's age and stemness score. The only significant exception was the uterus, where we observed an increased stemness with age. Moreover, we observed that stemness is positively correlated with cell proliferation and negatively correlated with cellular senescence. Finally, we also observed a trend that hematopoietic stem cells derived from older individuals might have higher stemness scores. In conclusion, we assigned stemness scores to human samples and show evidence of a pan-tissue loss of stemness during human aging, which adds weight to the idea that stem cell deterioration may contribute to human aging.

## INTRODUCTION

Although the aging process is the leading cause of human mortality and morbidity, being associated with several diseases, scientists still debate its causes and mechanisms [1–3]. Among the biological pathways associated with aging, we can highlight stem cell exhaustion, which argues that during normal aging, the decrease in the number or activity of these cells contributes to physiological dysfunction in aged tissues [4]. This concept is supported by the observation that aging is associated with reduced tissue renewal and repair at advanced ages [5]. Moreover, longevity manipulations in mice often affect growth and cell division, which has been hypothesized to relate to stem cells [6].

Despite their importance, *in vivo* detection and quantification of stem cells are challenging, which makes it difficult to study their association with aging, especially in humans [7]. In this context, detecting stemness-associated expression signatures is a promising strategy for studying stem cell biology. Stemness refers to a distinctive attribute marked by a series of molecular processes that delineate the essential properties of stem cells, enabling the generation of daughter cells and self-renewal. While widely employed in oncology, the exploration of this concept in gerontology has been comparatively limited [8–10].

In this study, we applied a machine learning method to detect stemness signatures from transcriptome data of healthy human tissues (see Methods). The methodology, developed by Malta et al. [9], was trained on stem cell classes and their differentiated progenitors and went through rigorous validation steps by Malta et al., including tests in several datasets from tumor and non-tumor samples. Although initially used to study oncogenic dedifferentiation, this approach has also been employed to study normal and pathological (non-tumorous) samples [11–15]. Therefore, we first downloaded expression data of 17,382 samples, divided into 30 tissues aged between 20 and 79 years, from GTEx in transcripts per million (TPM) [16]. After that, we followed the methodology by Malta et al. and assigned a stemness score to all GTEx samples [9]. Briefly, the stemness score varies from 0 (lowest stemness of the samples) to 1 (highest stemness of the samples). All the data generated are in Supplementary Table 1 and include the stemness score and clinical data from GTEx.

## RESULTS

**Figure 1:**
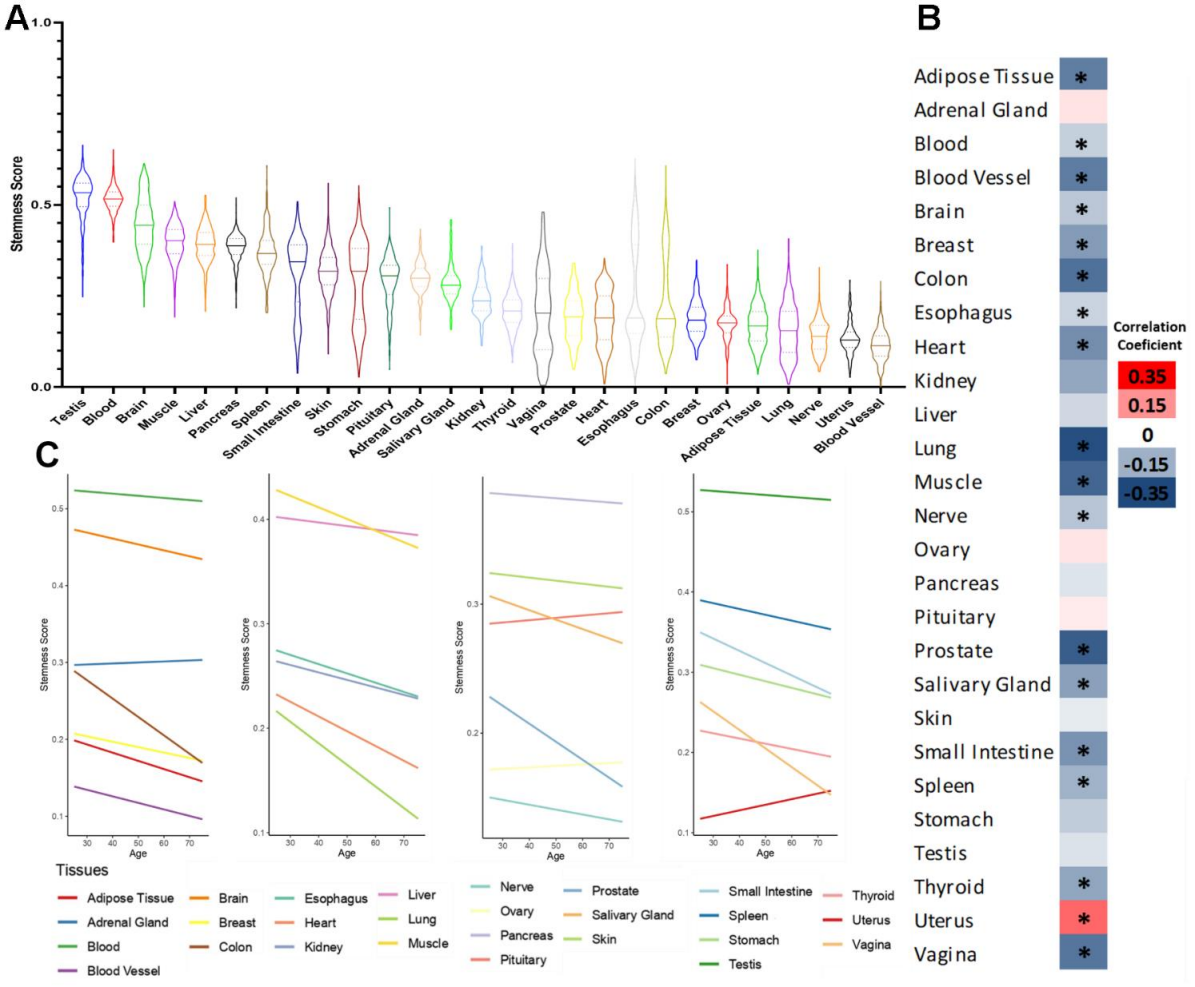
**Stemness levels during human aging.** (**A**) Distribution of stemness in human tissues. (**B**) Heatmap of Pearson’s correlation coefficient between stemness scores and age in human tissues. (**C**) Linear trend between stemness scores and age in human tissues. *FDR <0.05.

First, in Figure 1A, we show the distribution of stemness in the 27 human tissues with at least 50 samples. We observe that the highest stemness scores are in testis, which are known to have a higher number of stem cells [17]. Notably, blood ranks as the second tissue with the highest stemness. Even though most hematopoietic stem cells (HSC) are typically located in the bone marrow, a fraction can be identified in the bloodstream, presenting direct potential for clinical applications [18, 19].

Then, we analyzed the relationship between stemness and aging. In Figure 1B, we have a heatmap of the correlation coefficient (Pearson correlation test followed by Benjamini-Hochberg correction, Supplementary Table 2) between stemness score and age, and in Figure 1C, we show the linear trend of the same variables. We observe a pan-tissue loss of stemness in most tissues studied, with the only significant exception being the uterus. Interestingly, our group previously showed that the uterus also tends to behave differently concerning cellular senescence and gene expression signatures during aging [20, 21].

As death and sex can influence gene expression (and consequently the stemness score) [22], we conducted a linear model where we adjusted the stemness variation (log2FC) with the variables present in GTEx (see Methods for details). With this model, we observe a variation in stemness, which aligns with the direction of the age correlation results, indicating a decrease in stemness with age in most tissues (Supplementary Table 3). The only tissues that exhibit a different behavior are the liver and nerve, which, overall, corroborates our earlier observation.

**Figure 2:**
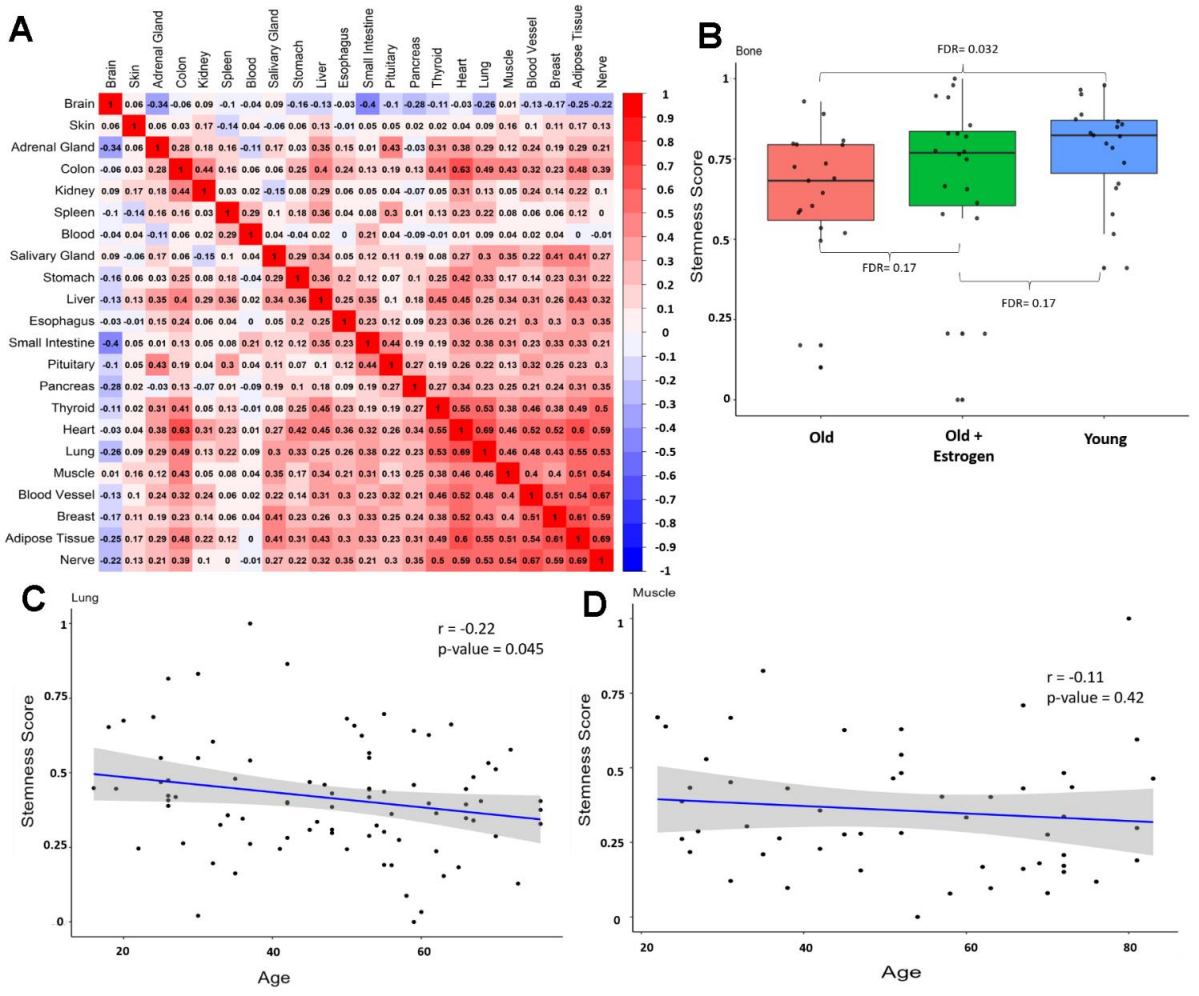
**Stemness in same individual and validation datasets.** (**A**) Correlation matrix between stemness across tissues from the same individual (excluding sex-related tissues) from GTEx. Numbers in each square represent the Pearson correlation coefficient. (**B**) Stemness levels of different groups in the alternative bone dataset, data from Weivoda et al. [30]. (**C**) Correlation between stemness and aging in an alternative lung dataset (*r* = -0.22, p-value = 0.045), data from Lee et al. [31]. (**D**) Correlation between stemness and aging in an alternative muscle dataset (*r* = -0.11, p-value = 0.42), data from Tumasian et al. [32].

Moreover, we constructed a correlation matrix (Figure 2A) to examine the association between stemness in different tissues from the same individual. Most correlations are positive, suggesting that stemness is a global attribute of the organism. A noteworthy exception is the brain, whose stemness exhibits a flat or negative correlation with other tissues.

To validate the GTEx results, we used additional datasets for lung, bone, and muscle (Figure 2B–2D). In bone, we observe a difference between the old and young groups (FDR = 0.032), and notably, women treated with estrogen replacement seem to have more stemness than the “natural” old group, suggesting that physiological variations (in this case, hormonal) can affect stemness (Figure 2B). Also, in lungs there is a significant decline in stemness with aging (Figure 2C) and a declining trend in muscle (Figure 2D). Taken together, these results indicate a trend toward a decline in stemness in human tissues during aging. Additionally, sample size appeared to be crucial in our analyses, and future studies should consider individual parameters, beyond age, that influence stemness.

**Figure 3:**
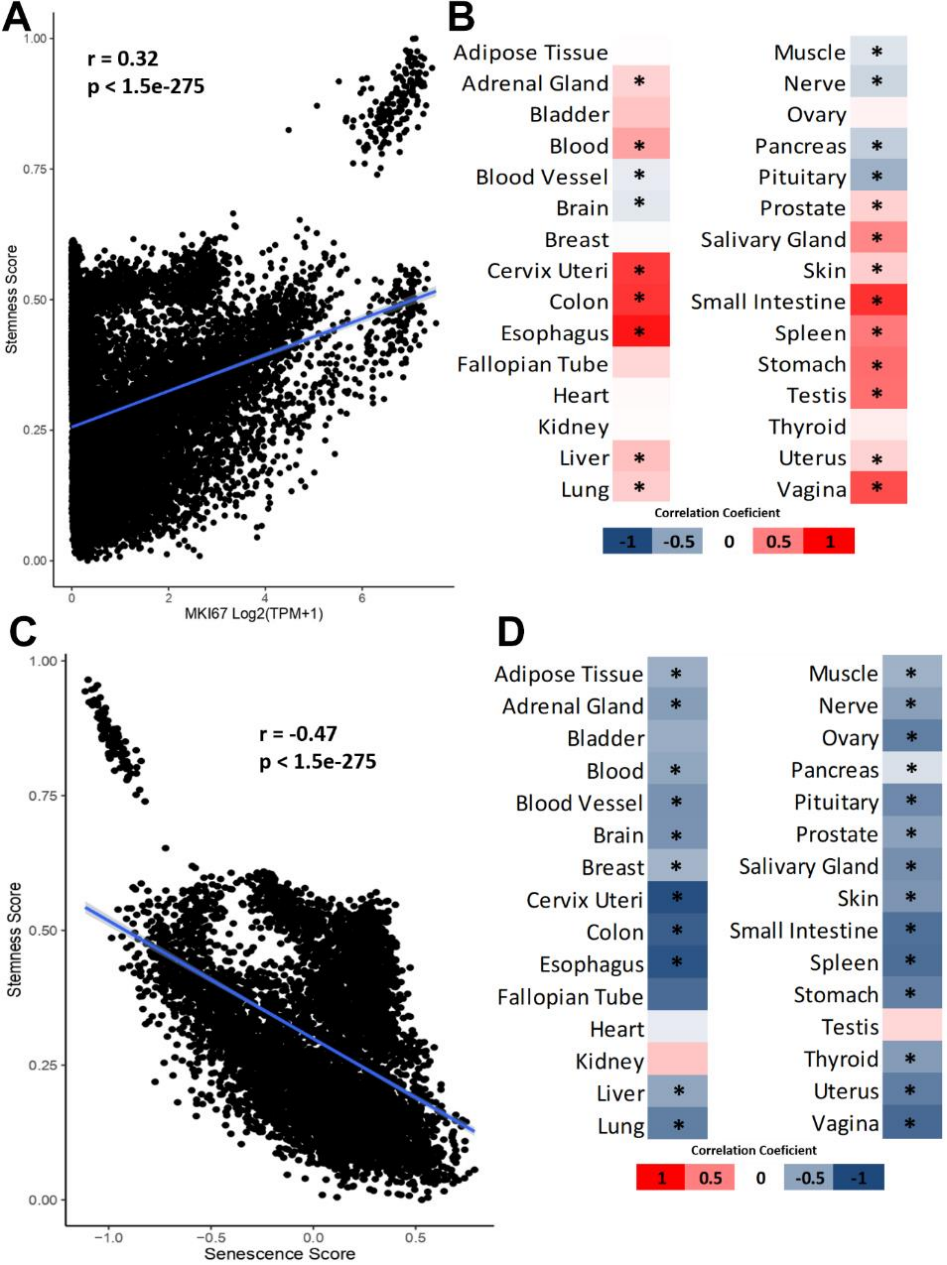
**Relationship between stemness, cellular proliferation and senescence.** (**A**) Correlation between stemness score and MKI67 expression. (**B**) Heatmap of Pearson’s correlation coefficient between stemness scores and MKI67 expression. (**C**) Correlation between stemness score and senescence scores. (**D**) Heatmap of Pearson’s correlation coefficient between stemness score and senescence score. *FDR <0.05.

To explore potential mechanisms, we associated stemness with cell proliferation and senescence, two processes associated with aging. We investigated the correlation between stemness and cell proliferation by acquiring gene expression data (in TPM) for the MKI67 proliferation marker from GTEx. Subsequently, we conducted a correlation analysis between the MKI67 expression and the stemness score of the samples. Figure 3A shows the correlation between stemness and proliferation in all GTEx samples (Pearson’s correlation test). Figure 3B shows a heatmap of the correlation coefficient in all tissues from GTEx (Pearson’s correlation test followed by Benjamini-Hochberg correction).

We observe a general positive correlation trend between stemness and cell proliferation, but this correlation is not observed in all tissues as exceptions include blood vessel, brain, muscle, nerve, pancreas, and pituitary results (Figure 3A, 3B). To validate this result, we employed the gene list provided in the study by Ramaker et al. [23]. In summary, the authors established and validated a proliferation index for healthy tissues and cells using the gene list from Venet et al. [24]. We correlated it with the stemness score using the average expression of all genes in the proliferation list, thereby obtaining a proliferation value per sample. The results reveal an even greater number of positive correlations, but still demonstrate negative and significant correlations, now specifically for the pancreas and pituitary (Supplementary Figure 1A). All the results of these correlations can be found in Supplementary Table 4. This suggests that, although cell proliferation is important for stemness, their relationship is complex.

Then, we used Wang et al. senescence score data to study the association between stemness and cellular senescence [25]. In total, we have 7123 samples with stemness and senescence values simultaneously, and the correlations were performed as before. We observe a negative correlation between stemness and senescence when considering all available GTEx samples (Figure 3C) and when we separate by tissue (Figure 3D) without any significant exception. Similar to the validation for cell proliferation, we employed the SenMayo genes to obtain an alternative senescence signature [26]. Subsequently, we correlated it with the stemness score (Supplementary Figure 1B). The results are similar, with all significant correlations between stemness and senescence being negative. All the results of these correlations can be found in Supplementary Table 5. These results indicate that although senescent cells and stem cells are not technically opposite states, they behave in opposite ways *in vivo* at the transcriptomic level.

Finally, GTEx samples come from bulk RNAseq containing multiple cell types. It is plausible that varying tissue types harbor distinct ratios of stem cells to somatic cells, thereby influencing the stemness score. However, the functional understanding of how stemness variation in stem cells concerning age remains elusive. Thus, it is uncertain whether there exists any age-related difference in the stemness attribute specifically within stem cells from older individuals. To address this uncertainty, we investigated whether the directly measured stemness in stem cells exhibits variation with age. To do so, we utilized data from Adelman et al. [27] and compared stemness levels directly in HSC isolated from two age groups: young (ages 18-30) and old (ages 65-75).

**Figure 4:**
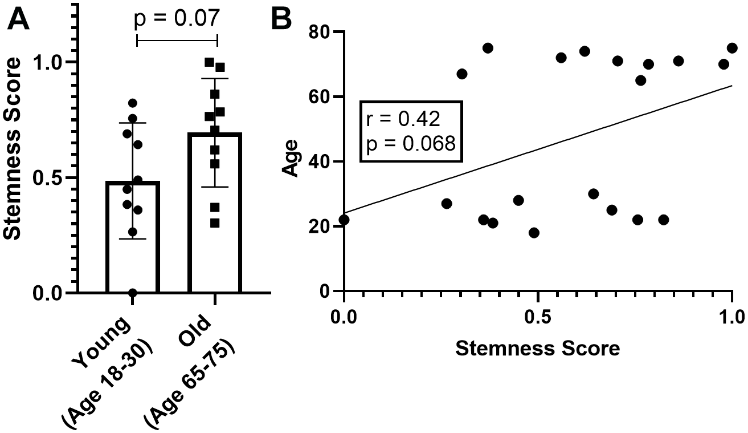
**Stemness levels in hematopoietic stem cells.** (**A**) Direct comparison between young and old groups. (**B**) Correlation between stemness score and age of the HSC donors.

We observed a quite unexpected result; despite not having a statistically significant difference, we see that both the comparison between the groups (Figure 4A, p=0.07) and the direct correlation (Figure 4B, r=0.42 and p=0.068), have an indicative trend of HSC from older donors to have more stemness. This result is interesting when we consider that literature shows evidence of increasing HSC numbers with age, which may suggest that age-related stem cell problems are functional rather than quantitative [27]. Nevertheless, more robust studies must be performed before we can draw more assertive conclusions.

## DISCUSSION

In this work, we studied the relationship between stemness and aging in humans. We found a loss of stemness in most aged tissues, which corroborates that stem cell exhaustion is important for aging. Although stem cell depletion in aging has already been observed in some tissues, such as satellite cells in the muscle [28], and hematopoietic stem cells in blood and bone marrow [29], as far as we know, we are the first to provide evidence of this in a pan-tissue manner.

We also show a positive trend between stemness and cell proliferation, but it is not global, with exceptions in some tissues. When we consider cellular senescence, we observe that the two phenomena are opposite in almost all tissues at the transcriptomic level.

It is important to note that many questions remain open. Our analyses are mainly correlation tests; therefore, we cannot determine cause and effect. This is important as we cannot assure whether the loss of stemness contributes to aging or is a cause or response of aging processes. Furthermore, we do not know whether the decrease in stemness a direct reduction of the stem cell pool is or refers to intrinsic characteristics of different cells in the tissue. In this sense, our HSC analyses corroborate a functional problem in aged stem cells, at least in blood stem cells. Ultimately, it is crucial to determine what drives these changes and which patterns and genes are associated with this process. Here is essential to highlight some papers that suggest that epigenetic modifications regulate stemness and could be a promising area for future studies [33, 34]. Additionally, our analyses suggest that stemness is a global attribute of the organism which correlates across tissues (except the brain), and future analyses may investigate how genetic polymorphisms contribute to individual stemness. Specifically, genome-wide association studies could be insightful in elucidating mechanisms associated with stemness. Additionally, the correlation of stemness among tissues could reflect systemic factors such as hormones (as suggested by the effect of estrogen in Figure 2B) or inflammation that impact cell proliferation [35, 36]. This presents three potential scenarios: the pan-tissue decrease in stemness may indicate either cell-intrinsic mechanisms, a systemic phenomenon, or a combination of both.

One limitation of our study is that it is based on a data-driven inference of stemness from transcriptomic signatures. Additionally, our method does not consider differences between types of stem cells in different tissues. Ideally, experimental validation will be necessary to corroborate our findings. Nevertheless, it is currently challenging to delineate an approach to measure stemness in aged human tissues experimentally, and thus we believe that our work can serve as a starting point for future research.

In conclusion, we provide the first evidence of a pan-tissue decrease of stemness during human aging and report an association between stemness and cell proliferation and senescence. This study also assigned a stemness score to more than 17,000 human samples, and these data can be useful for the scientific community for further studies.

## MATERIALS AND METHODS

### Transcriptome data from healthy tissues

RNA-Seq-based gene expression data from human tissues were downloaded from the GTEx portal (https://gtexportal.org) in transcripts per million (version 8) [16]. The raw RNA-Seq data were previously aligned to the human reference genome GRCH38/hg38 by the GTEx consortium.

We reduced the number of genes from the GTEx data to the same pairs found in the training matrix, i.e., we retained only the genes utilized by Malta et al. [9]. to calculate stemness. The training matrix is generated through a machine learning methodology (see below) and is the same as that in the original stemness paper by Malta et al. [9]. The resulting matrix contained 12,471 mRNA expression values and was used to calculate the stemness score. Here it is important to note that GTEx does not provide the actual age of each sample but rather age ranges (20-29, 30-39, 40-49, 50-59, 60-69, and 70-79). We then approximate the age of each sample to 25, 35, 45, 55, 65 and 75 years, respectively, as previously [20].

### Stemness score

To assign the stemness score of the GTEx samples we used a machine learning methodology built by Malta et al. [9].

In brief, Malta et al. built a predictive model using a one-class logistic regression on the pluripotent stem cell samples (ESC and iPSC) from the PCBC dataset [37–39]. The data were mean-centered, and the logistic regression was applied to the stem cell labeled samples to obtain the training signature. We then applied this signature to the GTEx transcriptome data, using Spearman correlations between the model’s weight vector and the sample’s expression profile. As a result, we have a stemness score for all GTEx samples ranging from low (zero) to high (one) stemness. Further details and validation of the methodology can be found in the original study [9].

Additionally, the highest stemness scores observed in Figure 1 (below 0.7) are due to the highest values >0.7 being associated with EBV-transformed lymphocyte samples, which are not represented in the figure. The GTEx data is segmented into sub-regions known as “tissue detail,” particularly within blood and skin, including cells derived from patients, fibroblasts, and lymphocytes transformed with EBV. Our study focuses on the overall stemness during aging, emphasizing major tissues without subdivisions. Cell lines from skin and blood were excluded in downstream analyses to prevent significant data distortion, although we calculated their stemness scores for potential use in future studies. Notably, transformed cells, expected to exhibit stem-like behavior compared to tissues, consistently display the highest stemness scores.

All this process was done using R and the code available on GitHub associated with the original paper [9]. We followed the author’s guidelines step by step.

### Linear model

To confirm that the stemness effect was not an artifact resulting from the interference of cause of death, sex, or tissue subregion, we applied a linear model similar to what we had done previously [40].

For each tissue, fold change with age was calculated using the model below. If any variable is not present in the tissue (e.g., sex for vagina or region for thyroid), it is disregarded in the analysis. All information on the subjects was taken directly from the GTEx portal (https://gtexportal.org/home/datasets).


$${\text{Y}}_{\text{\_ij}}=\alpha {\text{Age\_}}_{\text{i}}+\beta {\text{Sex\_}}_{\text{i}}+\gamma {\text{Death\_}}_{\text{i}}+\delta {\text{Region}}_{\text{i}}+\varepsilon {\text{\_}}_{ij}$$

The variables are defined as follows:

- *Y__ij_*: The stemness score *j* in sample *i*.
- *Age__i_*: The age of sample *i* – continuous variable.
- *Sex__i_*: The sex of sample *i –* categorical variable.
- *Death__i_*: The death classification of sample *i* based on the 4-point Hardy scale *–* categorical variable [41].
- *Region__i_*: The tissue region cells were extracted from for sample *i –* categorical variable.
- ε_*_ij_*: The error term for stemness score *i* in sample *j*.

Linear model was generated using the R package limma, using the lmFit() function [42, 43].

### Correlation matrix

To assess the stemness behavior between tissues across the same individual, we constructed a correlation matrix using Pearson coefficients. For the few cases in which a specific subject had two or more samples from the same tissue, we used the average stemness score to calculate the correlation matrix.

For the Figure 2, we employed the R package ‘corrplot’ with hierarchical clustering option [44]. In this analysis, we excluded sex tissues (testis, prostate, vagina, uterus, and ovary) as they cannot be correlated with each other and would interfere with the hierarchical classification. In Supplementary Figure 2, we included the correlation matrix with all GTEx samples with more than 50 samples, without clustering.

### Validation datasets

For validation, we selected three datasets on the aging of healthy tissues. For lung data, we utilized the dataset from Lee et al. [31], which comprises 86 samples with an age range of 16 to 76 years. For muscle data, we utilized the dataset from Tumasian et al. [32] which comprises 53 samples with an age range of 22 to 81 years. The bone data is from Weivoda et al. [30] and consists of 58 female samples divided into three groups: 19 young women (30.3 ± 5.4 years), 19 old women (73.1 ± 6.6 years), and 20 old women treated with 3 weeks of Estrogen therapy (70.5 ± 5.2 years).

All datasets are from bulk RNA-seq, like GTEx. Additionally, the stemness score was calculated independently for each tissue, following the same procedure as in the GTEx analysis. For correlations, we used the Pearson coefficient, and for the analysis of bone data, we employed the Kruskal-Wallis test.

### Correlation with senescence and cell proliferation

To correlate the stemness score with cell proliferation, we initially retrieved the expression data for the proliferative marker MKI67 from the GTEx database in TPM (transcripts per million). To validate this result, we employed the gene list provided in the study by Ramaker et al. [23]. In summary, the authors established and validated a proliferation index for healthy tissues and cells using the gene list from Venet et al. [24]. The gene list for constructing the proliferation index was identified from the top 1% of genes most positively correlated with the proliferation marker PCNA (proliferating cell nuclear antigen) across 36 healthy tissues. We applied the same gene list and calculated the average expression of all genes within the proliferation list, thus obtaining a proliferation value per sample for correlation with the stemness score.

For the correlation with cellular senescence, we utilized the study by Wang et al. [25], which calculated a senescence score for GTEx samples. Again, for validation, we employed the SenMayo gene list [26], one of the most used by the scientific community for senescence studies. We employed the same strategy as before, using the average expression of SenMayo genes to correlate with the stemness score.

### Analysis of hematopoietic stem cells

We first downloaded normalized counts of bulk RNA-seq from the work of Adelman et al. [27]. In this study, the authors isolated human hematopoietic stem cells from two groups with ten samples each: young (age 18-30) and old (age 65-75). We applied the same approach as above to generate stemness scores in these samples. We then compared stemness with age by direct comparison between the two groups (Student’s t-test) and by Pearson correlation. The graphs for this analysis were constructed using GraphPad Prism 8.

### Statistical analysis and graphs

We applied Pearson correlations to all analyses performed in this work using R functions. In tissue-specific analyses, the p-value was corrected using Benjamini-Hochberg’s FDR methodology. All correlations coefficients, p-values and FDR are presented in Supplementary Tables 2, 4, 5.

Linear trends and the respective graphs were built using the ggplot2 (version 3.3.6) with standard parameters [45]. Heatmaps and violin plot were built using Microsoft Excel and GraphPad Prism 8, respectively.

## Supplementary Material

Supplementary Figures

Supplementary Table 1

Supplementary Tables 2 and 3

Supplementary Table 4

Supplementary Table 5
