# Supplementary material for: Evidence of a pan-tissue decline in stemness during human aging
Source: Aging (Albany NY). 2024 Apr 4;16(7):5796–810. doi: 10.18632/aging.205717 (PMC11042951; doi:10.18632/aging.205717)
Supplement: Supplementary Figures [file aging-16-205717-s001.pdf]

SUPPLEMENTARY FIGURES

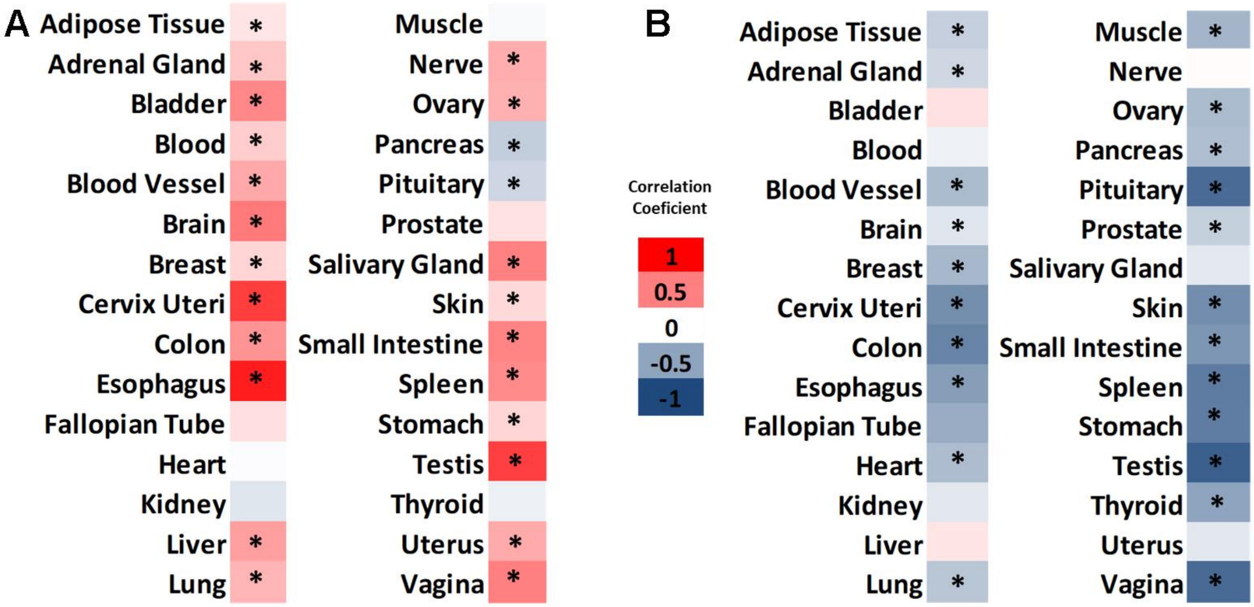

Supplementary Figure 1. Validation of the relationship between stemness and (**A**) cellular proliferation and (**B**) and cellular senescence. (**A**) Heatmap of Pearson's correlation coefficient between stemness scores and alternative proliferation signature expression. (**B**) Heatmap of Pearson's correlation coefficient between stemness score and alternative senescence signature. *FDR <0.05.

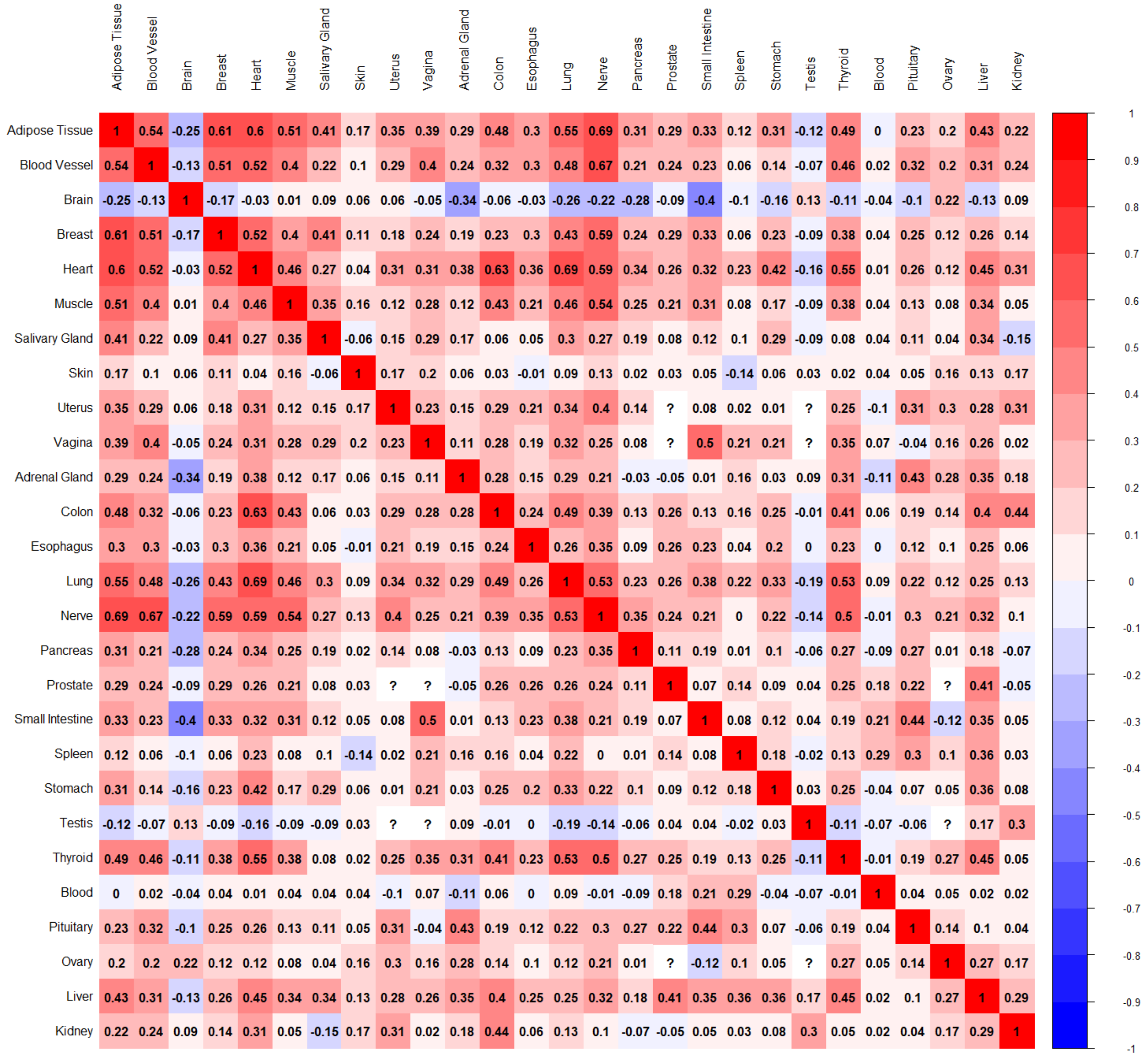

Supplementary Figure 2. Stemness in same individual. Correlation matrix between stemness across tissues from the same individual including all from GTEx with more than 50 samples.
