# Supplementary material for: Evidence of a pan-tissue decline in stemness during human aging
Source: Aging (Albany NY). 2024 Apr 4;16(7):5796–810. doi: 10.18632/aging.205717 (PMC11042951; doi:10.18632/aging.205717)
Supplement: Supplementary Tables 2 and 3 [file aging-16-205717-s003.pdf]

Supplementary Table 2. Pearson correlations between age and stemness for GTEx tissues.

Tissues	Pearson correlation coefficient	p-value	FDR
Adipose Tissue	-0.248268542414925	2.27231621430602E-18	3.18124270002843E-17
Adrenal Gland	0.0384114134930505	0.539076922689334	0.603766153412054
Blood	-0.105536139695034	0.00369428913248449	0.00795693043919735
Blood Vessel	-0.248528478217365	3.05159497445873E-20	8.54446592848446E-19
Brain	-0.105885813817273	4.88796994153834E-08	1.95518797661534E-07
Breast	-0.189833529663225	0.0000425392948300989	0.000119110025524277
Colon	-0.272586921831837	9.71476709902331E-15	5.44026957545305E-14
Esophagus	-0.0816728170697093	0.00188902636307627	0.00440772818051129
Heart	-0.215461374796781	1.67812727186664E-10	7.83126060204432E-10
Kidney	-0.168498753029081	0.114462049422107	0.152616065896143
Liver	-0.076337893152415	0.253073848569264	0.322093989088154
Lung	-0.337032351467181	8.06965764103894E-17	5.64876034872726E-16
Muscle	-0.293365940400865	2.10797704472822E-17	1.96744524174634E-16
Nerve	-0.109767991492994	0.00626193249699757	0.0116889406610621
Ovary	0.0345375996208705	0.645315465398801	0.669216038191349
Pancreas	-0.0509929330670782	0.357262097175113	0.416805780037631
Pituitary	0.0283488369833193	0.634868254840594	0.669216038191349
Prostate	-0.307700492095631	9.04657400634443E-07	3.16630090222055E-06
Salivary Gland	-0.181185493557832	0.0210331674059784	0.034642863962788
Skin	-0.0525400987836706	0.0577630962732517	0.0851245629290026
Small Intestine	-0.205803381141574	0.00471683432615062	0.00943366865230124
Spleen	-0.146000484074378	0.0233955019870618	0.036393003090985
Stomach	-0.0963926323141464	0.0681127327707073	0.0953578258789902
Testis	-0.0546348003178722	0.300559430729044	0.365898437409271
Thyroid	-0.170971973225073	0.0000111792150896284	0.0000347797802788441
Uterus	0.207810683597776	0.0130788012085986	0.0228879021150475
Vagina	-0.262016682832725	0.000952517994451209	0.00242459125860308

Supplementary Table 3. Linear model to verify the variation of stemness in GTEx tissues.

Tissue	Log2FC per year	Log2FC in 50 years	p-value	FDR
Adipose Tissue	-0.000508871204838363	-0.0254435602419182	0.0000494425970939582	0.000266990024307374
Adrenal Gland	0.000295360042929763	0.0147680021464882	0.181502652845311	0.278955027354782
Blood	-0.000141231699575227	-0.00706158497876136	0.185970018236521	0.278955027354782
Blood Vessel	-0.000601285654920596	-0.0300642827460298	3.23746957147567E-11	4.37058392149215E-10
Brain	-0.000628745864324055	-0.0314372932162028	2.16910016169668E-11	4.37058392149215E-10
Breast	-0.000506588806626808	-0.0253294403313404	0.00545773601090029	0.0147358872294308
Colon	-0.000854297051968771	-0.0427148525984386	0.00118111702771642	0.00354335108314926
Esophagus	-0.00043577820067519	-0.0217889100337595	0.000129658302130164	0.000583462359585739
Heart	-0.00046301311509361	-0.0231506557546805	0.00693564269220297	0.0170238502444982
Kidney	-0.000504848254014379	-0.0252424127007189	0.289037261515252	0.390200303045591
Liver	0.000235805896736568	0.0117902948368284	0.430834126117321	0.528750972962167
Lung	-0.00117195427280499	-0.0585977136402496	2.61622509565382E-07	2.35460258608844E-06
Muscle	-0.000550957952615605	-0.0275478976307802	0.0000275044497831532	0.000185655036036284
Nerve	0.0000554026984020488	0.00277013492010244	0.698425368638435	0.734710953179775
Ovary	0.000103215787604041	0.00516078938020204	0.707499436395338	0.734710953179775
Pancreas	-0.0000489635447119748	-0.00244817723559874	0.784548643441908	0.784548643441908
Pituitary	0.000183487921132126	0.00917439605660631	0.637845924778307	0.734710953179775
Prostate	-0.00105720626082543	-0.0528603130412714	0.000357694339715051	0.00137967816747234
Salivary Gland	-0.000558755182858346	-0.0279377591429173	0.108616403106773	0.183290180242679
Skin	-0.000169454348560241	-0.00847271742801207	0.278673896259403	0.390200303045591
Small Intestine	-0.00130587456242865	-0.0652937281214325	0.0149427618794	0.03362121422865
Spleen	-0.000538020904220044	-0.0269010452110022	0.0885993454626721	0.15947882183281
Stomach	-0.000181483941126939	-0.00907419705634696	0.6834666678237	0.734710953179775
Testis	-0.000537220018057982	-0.0268610009028991	0.0361769919050425	0.0697699129597249
Thyroid	-0.000127449447276679	-0.00637247236383397	0.392622754594174	0.504800684478224
Uterus	0.0010355733855643	0.051778669278215	0.000566271087020793	0.00191116491869517
Vagina	-0.00160212723077951	-0.0801063615389757	0.0269868515112603	0.056049614677233
